# Understanding Vaccine Perceptions and Willingness to Receive COVID-19 Vaccination: Opportunities to Strengthen Public Health Responses and COVID-19 Services for People Who Use Drugs

**DOI:** 10.3390/vaccines10122044

**Published:** 2022-11-29

**Authors:** Ashly E. Jordan, Rwaida Izar, Renée Nicolas, Nisha Beharie, Alex Harocopos

**Affiliations:** Bureau of Alcohol and Drug Use Prevention, Care and Treatment, New York City Department of Health and Mental Hygiene, 42-09 28t St, Floor 19, Queens, New York, NY 11101, USA

**Keywords:** vaccination, viral infections, public health programming, people who use drugs, prevention, community health, COVID-19

## Abstract

Background: People who use drugs (PWUD) are at high risk for COVID-19 infection, morbidity, and mortality. COVID-19 vaccines are safe and effective at reducing serious illness and death from COVID-19. There are sparse data on the perceptions and willingness of PWUD to receive COVID-19 vaccination. Materials and Methods: In order to assess the perceptions of, and willingness to receive, COVID-19 vaccination among PWUD, we conducted a rapid survey-based assessment of 100 PWUD in NYC (Spring 2021) who reported not having received COVID-19 vaccination and who reported past 30-day illicit drug use. Results: More than 80% of respondents agreed that personally receiving a COVID-19 vaccine was important for the health of others in the community, and endorsing this belief was significantly associated with COVID-19 vaccine willingness reflecting a high prevalence of altruistic beliefs (*p*-value: 0.01). Other reported perceptions that were significantly associated with COVID-19 vaccine willingness were believing that COVID-19 vaccines are safe for PWUD and trusting COVID-19 information from their healthcare providers (*p*-values < 0.05). That said, 62% reported being unwilling to receive a COVID-19 vaccine, and 70–83% had concerns about general vaccine safety/efficacy. Examining pairs of questions to explore potential ambivalence between vaccine endorsement and vaccine concerns identified that 56–65% simultaneously reported vaccine safety/efficacy concerns and beliefs that vaccination was an important intervention. Of the 75 respondents who reported past 30-day use of harm reduction and/or substance use disorder (SUD) programs, nearly 90% reported these programs as trusted sources of COVID-19 information. Conclusion: Most participants reported altruistic beliefs about the role of vaccines for community health, including COVID-19 vaccines, and this altruism was associated with willingness to be vaccinated against COVID-19. These findings suggest a complex relationship between beliefs about the role of vaccination in community health and the safety/efficacy of vaccines; this ambivalence suggests that COVID-19 vaccine willingness may not be firmly fixed, indicating potential opportunities to address questions and build vaccine confidence. Harm reduction and SUD programs could be leveraged to further engage PWUD in receipt of COVID-19 information and/or vaccination. Recognizing vaccine ambivalence, emphasizing collective and individual benefits of vaccination, and messaging from trusted sources may be promising approaches to increase vaccination in this population.

## 1. Introduction

COVID-19 is a multisystem disease caused by the highly transmissible SARS-CoV-2 virus [1,2,3]. After emerging globally in late 2019, COVID-19 was declared a pandemic in March 2020 by the World Health Organization and New York City (NYC) was subsequently identified as the initial epicenter of the US epidemic [1,2,3]. As of September 2022, 29% of NYC residents have been diagnosed with COVID-19 infection and there have been 184,000 and 41,803 COVID-19-related hospitalizations and deaths, respectively; yet, recent national seroprevalence estimates suggest that overall COVID-19 infection rates may be even higher [4,5]. In NYC, Non-Hispanic Black and Hispanic New Yorkers have died of COVID-19 at twice the rate of White New Yorkers [4,5]. Further, national data delineate socio-demographic and geographic gradients by income for COVID-19 case rates, hospitalizations, and death, including among people who use drugs (PWUD), where increased risk operates through pathways such as labor-market exposure (workforce characterized by “essential workers”), racial segregation, and housing conditions [6,7,8,9,10,11,12,13,14,15,16].

In the US, the COVID-19 pandemic emerged during the Trump administration, a time period characterized by policies which posed a threat to public health through the politicization and repudiation of science, inciting racialized and nativist violence (e.g., surveillance, policing), undermining health insurance coverage, weakening social support systems, and shifting public spending away from public health to other purposes such as military spending and tax windfalls for corporations and the wealthy [17]. These have directly and indirectly contributed to the pandemic’s impact among specific populations and areas in the US [18,19].

Racial and ethnic disparities in COVID-19 case rates, hospitalizations, and deaths reflect historic and ongoing structural racism, resulting in inequities in access to prevention and health care and in vaccine-preventable morbidity and mortality from other infections such as influenza and pneumonia [20,21,22,23,24,25]. PWUD and people with substance use disorders (SUD) are additional populations rendered more vulnerable through similar mechanisms of structural oppression [7,26,27,28]. PWUD are both more likely to become infected with COVID-19 and to have higher rates of COVID-19-associated hospitalization and death than the general population; among PWUD, racial and ethnic disparities in COVID-19-related outcomes reflect disparities in the general US population [8,10,29,30,31]. Further, even among those vaccinated in the past 12 months, PWUD are more likely to be re-infected with COVID-19, reflecting both the need to ensure that PWUD complete a primary vaccine series, and the need to ensure attention to aspects of their social and structural context which could otherwise be barriers to vaccination [7,17,28,29,32,33].

Globally, as of early 2022, COVID-19 vaccination has contributed to the prevention of more than 14 million cases, 1 million hospitalizations, and a quarter of a million deaths. [34] Historically, vaccination has been a critical population-, social-, and individual-level public health intervention that has led to the eradication, elimination, and control of a range of bacterial and viral infections [35,36]. In recent decades, vaccine refusal has become a serious threat to public health, contributing to resurgences of measles and pertussis and to delays in the global elimination of polio [37]. Delays in vaccination have contributed to “vaccination cold spots” where local outbreaks continue to occur, and in which variants can propagate and have adverse subsequent effects on vaccine efficacy [38]. Further, data indicate that COVID-19 vaccine access may not be the strongest predictor of vaccination uptake, as various studies have identified that willingness to be vaccinated against COVID-19 is strongly associated with subsequent vaccine uptake [39,40].

In addition, experiences of stigmatization and discrimination based on race, ethnicity, and/or being identified as a PWUD contribute to medical mistrust and may decrease vaccine confidence [41,42,43]. PWUD can experience stigma and discrimination both broadly within society and by providers and health systems, based on their identification as a PWUD or based on internalized stigma, which can create or reinforce medical mistrust and result in worse health outcomes [7,44,45,46]. Stigma and discrimination may lead PWUD to less frequently engage, have more mistrust in care and prevention systems, and more frequently accept or be influenced by misinformation [33,47,48].

Complex and interacting factors have contributed to a lack of confidence in COVID-19 vaccines and the role of vaccination in COVID-19 control [49,50,51]. Anti-vaccination misinformation (i.e., unintentionally false or confusing information) and disinformation (i.e., deliberately misleading or false information, “propaganda”) have increased in recent decades, having been facilitated in part through the mass media in formats such as television, the internet, social media, and other “news” platforms; social media contributes to the ease with which misinformation and disinformation can be propagated in the COVID-19 pandemic [52,53,54]. Unless effectively countered, misinformation and disinformation can undermine confidence in safe and efficacious vaccines [33,47,48].

A range of individual, social, and structural factors have been found to improve vaccination rates, including higher education levels, more accurate risk perception, receiving accurate information from trusted sources, and knowing trusted or respected individuals who have been vaccinated [55,56,57,58]. Structural factors include ensuring access to vaccination through the use of convenient and non-stigmatizing settings; for example, syringe services programs (SSPs), harm reduction programs, and drug treatment programs have been valuable sites for providing health information and other (non-COVID-19) vaccinations to PWUD [59,60,61,62,63].

In this study, the NYC Department of Health and Mental Hygiene (DOHMH)’s Bureau of Alcohol and Drug Use Prevention, Care, and Treatment sought to assess the knowledge, experience, and perceptions regarding vaccination in general, and COVID-19 vaccination specifically, among PWUD who had not yet been vaccinated for COVID-19.

## 2. Materials and Methods

### 2.1. Study Context

The survey was conducted in NYC in May–June 2021, approximately 14 months after the COVID-19 outbreak was declared a pandemic by the World Health Organization in March 2020 and NYC became the epicenter of the pandemic [64,65]. By this time, COVID-19 vaccinations (i.e., two mRNA vaccines: ‘Pfizer’ and ‘Moderna’, and one viral vector vaccine: ‘Johnson and Johnson’) had been found to be efficacious and effective in trials and granted emergency use authorization from the U.S. Food and Drug Administration (FDA) [66]. Further, vaccine access at the time the survey was administered was characterized by an initial period in which COVID-19 vaccine eligibility was prioritized for those most at-risk of severe illness and was later expanded to include all adults. Vaccination scheduling systems initially were complex and time consuming, which contributed to inequities in access and disadvantaged those without computer or internet access, the skills to navigate the online scheduling system, or ready access to transportation [67,68].

### 2.2. Study Population and Sampling

This assessment (henceforth referred to as “the survey”) was conducted as part of the Rapid Assessment and Response (RAR) team’s initiative to assess PWUDs’ experiences with and perceptions of vaccination in general and COVID-19 vaccination specifically in order to directly inform COVID-19 vaccine messages and public health programs. The RAR team routinely conducts community-based work among PWUD and is in frequent communication with SSPs to identify areas where PWUD congregate.

The study team has extensive experience in engaging PWUD using street-based methods, having received professional training in street-based recruitment methods and conducted street-based recruitment for public health research collectively for more than 10 years. Potential participants were identified via street-based recruitment in areas of four NYC boroughs (the Bronx, Brooklyn, Manhattan, and Queens). Survey staff were working with limited resources and time given the active COVID-19 public health emergency at the time of the survey; thus, the convenience sample was recruited from four out of the five NYC boroughs.

Eligibility included age ≥ 18 years, reported use of illicit drugs in the past 30 days, and not having received any dose of a COVID-19 vaccine. Surveys were conducted in both English and Spanish by bilingual study team members.

Of 144 potentially eligible individuals approached, 43 were ineligible due to not having used illicit drugs in the prior 30 days (n = 17), already having been vaccinated against COVID-19 (n = 12), or both (n = 14). Surveys were conducted by study staff and responses were input to REDCap using a tablet or laptop computer in real time. Participants were provided gift card incentive (USD 25) in gratitude for their time and for sharing person information about their lives and their experiences.

### 2.3. Survey Development

The survey was developed using validated items from prior surveys available from the PhenX Toolkit (maintained by RTI International and provides recommended standard data collection protocols and items for conducting biomedical research). Considering that both COVID-19 and COVID-19 vaccination were both new and rapidly evolving, several of the survey items used were adapted from prior surveys regarding vaccine access, beliefs, and experiences for other vaccine-preventable conditions such as hepatitis B virus and influenza.

An Agency staff member who is a native Spanish speaker translated the survey from English into Spanish using recommended Agency standards. We then had a second staff member who is a native Spanish speaker test the survey for accuracy. Any discrepancies were raised and resolved among our bilingual English–Spanish speaking team.

### 2.4. Study Domains and Variables

The survey focused on PWUDs and (a) their perceptions of and experiences with vaccination in general, including questions on the role of vaccination in community health; (b) sources, use of, and confidence in information about COVID-19; (c) perceptions of COVID-19 and COVID-19 vaccination; and (d) willingness to receive a COVID-19 vaccine.

The primary outcome of this analysis was the survey question “If you were offered COVID-19 vaccination right now, would you get it?” with the binary response category of yes or no.

### 2.5. Data Analysis

Univariate descriptive statistics were conducted for the overall survey population and were stratified by COVID-19 willingness. Bivariate analyses examining relationships between COVID-19 vaccine willingness and a range of socio-demographic factors and key thematic areas (as described above) were conducted using the chi-square and Fisher’s exact tests. Further, we examined several pairs of questions to assess overlapping responses endorsing statements that were seemingly contradictory (e.g., simultaneously endorsing the statement “vaccination is good for the health of one’s community” and “pharmaceutical companies cover up the dangers of vaccines”). We calculated measures of significance when statistically feasible. Analyses were conducted using R software (version 4.0.3; the R Foundation).

## 3. Results

Of the 101 eligible individuals, 100 (99%) participated; the demographics of participating PWUD are presented in Table 1. Almost half (46%) of the interviews were conducted in the Bronx. Participants were mostly male (80%) and nearly 44% self-identified as Hispanic, with 68% of Hispanic participants reporting Puerto Rican ancestry. Most participants were between the ages of 30 and 59 years (90%). One-third of participants reported having received services from SSPs in the past 30 days.

### 3.1. COVID-19 Vaccine Willingness

Among the 100 participants, 62 (62%; 95% CI: 61–63%) were not willing to receive a COVID-19 vaccine if offered. Non-Hispanic White participants more frequently reported being unwilling to receive COVID-19 vaccine (21/30, 70%) compared to non-Hispanic Black participants (11/22, 50%) and Hispanic participants (28/44, 64%), although these findings were not statistically significant (Table 1). Willingness to receive a COVID-19 vaccine increased with age (*p*-value: 0.024) (Table 1). Those ≥40 years of age were more likely to be willing to receive a COVID-19 vaccine than those <40 years of age (68/100 vs. 32/100, *p*-value: <0.01) (Table 1). Participants reporting that they would feel more comfortable getting a COVID-19 vaccine if other people they respected received it first (65/100, 65%) were significantly more likely to express willingness to be vaccinated against COVID-19 (29/38 (78%) vs. 36/62 (58%), *p*-value: 0.04) (Table 2).

Overall, 30% (95% CI: 28–32%) of those who reported being willing to receive a COVID-19 vaccine were not very confident in that decision and 40% (95% CI: 38–42%) of those who reported not being willing to receive a COVID-19 vaccine were not very confident in that decision.

Statements on adverse effects and vaccine safety indicated that while 83 (83%) reported being concerned about potential serious adverse effects of COVID-19 vaccines, this was not significantly associated with willingness to receive a COVID-19 vaccine (28/83 (34%) vs. 10/17 (59%), *p*-value: 0.052) (Table 3). Over two-thirds agreed with statements that vaccine efficacy data are often fabricated (67%), that people are deceived about vaccine safety (77%), and that pharmaceutical companies and governments cover up dangers posed by vaccines (82% and 74%, respectively); agreement with any of these statements was associated with reduced willingness to be vaccinated against COVID-19 (all *p*-values: <0.01) (Table 3).

### 3.2. Perceived COVID-19 Vaccine Safety among Specific Communities

A majority of participants (80%) agreed that COVID-19 vaccination was safe for people of their race/ethnicity including 20/22 (91%) participants who identified as non-Hispanic Black, 36/44 (82%) who identified as Hispanic, and 20/30 (67%) who identified as non-Hispanic White (data not shown). Overall, those who agreed that COVID-19 vaccines were safe for people of their race/ethnicity were more willing to be vaccinated against COVID-19 (36/80 (45%) vs. 2/20 (10%), *p*-value: <0.01). Participants who thought that COVID-19 vaccines were safe for PWUD were more willing to receive COVID-19 vaccination (36/78 (46%) vs. 2/22 (9%), *p*-value: <0.01) (Table 3). Small subgroup sample sizes precluded examination of associations between responses to this question and willingness to be vaccinated against COVID-19.

### 3.3. Vaccination and the Health of Others

Overall, 83 (83%) of participants agreed that vaccines in general were important for the health of their community, with 20/22 (91%), 38/44 (86%), and 21/30 (70%) of non-Hispanic Black, Hispanic, and non-Hispanic White participants, respectively, agreeing with this statement (Table 2). Fewer non-Hispanic White participants thought that vaccines were important for the health of their community than those of all other race/ethnicities combined (21/30 (70%) vs. 62/70 (89%), *p*-value: 0.048). Agreeing that vaccination in general was good for the health of one’s community was significantly associated with an increased willingness to receive a COVID-19 vaccine (36/83 (43%) vs. 2/17 (12%), *p*-value: 0.02) (Table 3). Small race/ethnicity subgroup sample sizes precluded examination of associations between responses to this question and willingness to be vaccinated against COVID-19.

Overall, 82 (82%) of participants agreed that personally receiving a COVID-19 vaccine was important for the health of others in the community, including 20/22 (91%), 39/44 (87%) and 20/30 (67%) of non-Hispanic Black, Hispanic, and non-Hispanic White participants, respectively. Agreeing that having oneself vaccinated against COVID-19 was important for the health of others in one’s community was significantly associated with willingness to be vaccinated (36/38 (95%) vs. 46/62 (74%), *p*-value: 0.01) (Table 3). Significantly fewer non-Hispanic White participants thought that having oneself vaccinated against COVID-19 was important for the health of others in one’s community compared to those of all other races/ethnicities combined (20/30 (67%) vs. 62/70 (89%), *p*-value: 0.02). Small subgroup sample sizes precluded examination of associations between responses to this question and willingness to be vaccinated against COVID-19.

### 3.4. Exploring Potential Ambivalence

We examined several pairs of questions to explore potential ambivalence about COVID-19 vaccination by identifying the proportion of participants who simultaneously endorsed apparently conflicting responses (Table 2). A majority of participants simultaneously endorsed apparently conflicting statements regarding concerns about vaccine safety and efficacy and statements about the important role of vaccination in community health. For example, 82 (82%) of participants believed that pharmaceutical companies cover up the dangers of vaccines and 83 (83%) agreed that vaccines are important for the health of their communities, with 65% of participants simultaneously holding both beliefs (Table 2).

### 3.5. Information Sources

Participants reported receiving most of their COVID-19 vaccine-related information from family and friends (26%), TV, (25%), social media (12%), and healthcare providers (18%). A majority of participants reported low or no confidence in information about COVID-19 received from TV and social media. Of participants who reported past 30-day use of harm reduction and SUD programs (75%), 89% and 79%, respectively, considered them to be trusted sources of COVID-19 information. While only 18% reported receiving COVID-19 information from a healthcare provider, almost 72% of these respondents trusted this source. Overall, those who reported being confident in their healthcare providers as sources of COVID-19 vaccine information were significantly more likely to be willing to be vaccinated against COVID-19 (32/72 (44%) vs. 4/22 (18%), *p*-value: 0.03). Small race/ethnicity subgroup sample sizes precluded examination of associations between responses to these questions and willingness to be vaccinated against COVID-19.

### 3.6. COVID-19 Vaccine Access

Among those who reported having attempted to make an appointment to receive a COVID-19 vaccine (n = 23, 23/100, 23%), six (6/23, 26%) had not received one because they were not able to find an appointment slot, nine had an appointment scheduled for the future, and seven had an appointment scheduled in the past that they ultimately did not attend. Participants reported that the three most important factors facilitating COVID-19 vaccine access were vaccine sites close to where one lives or stays (46/100, 46%), flexible drop-in hours (34/100, 34%), short wait times (34/100, 34%), and free or low-cost transportation to and from the vaccination site (32/100, 32%).

## 4. Discussion

A majority of participants reported altruistic beliefs about the role of vaccines, including COVID-19 vaccines, in community health. Previous research has demonstrated an association between altruism among PWUD and reduced HIV-related risk behaviors [69,70,71]. Consistent with our findings, a recent systematic review and meta-analysis identified a desire to protect the health of others as one of the most potent factors contributing to willingness to receive COVID-19 vaccination globally [40,72,73]. Among our participants, those who believed that COVID-19 vaccines were safe (a) for people of their race and/or ethnicity and (b) for other PWUD were more likely to be willing to receive a COVID-19 vaccine. This finding suggests that emphasizing vaccine safety within social and/or drug use networks can potentially facilitate receptivity to vaccination. COVID-19 vaccination messages and public health responses that emphasize the collective benefits of an individual’s decision to be vaccinated may hold promise to increase vaccine acceptance among PWUD.

Forty percent of those who reported not being willing to receive COVID-19 vaccination said they were not confident in this decision, suggesting an opportunity to engage PWUD regarding their concerns about the COVID-19 vaccines, including the role that respected people in trusted settings may have in strengthening COVID-19 vaccine confidence. As has been found in other recent studies, participants expressed concerns about potential serious adverse effects of COVID-19 vaccines, the validity of vaccine safety data, and transparency about vaccine risks by pharmaceutical companies and government officials [72,73,74]. Yet, most PWUD simultaneously endorsed statements agreeing with the importance of vaccines for community health and agreed that COVID-19 vaccines were safe for their racial/ethnic group(s) or for other PWUD. Further, a majority reported that they would feel more comfortable being vaccinated against COVID-19 if other people they respected were vaccinated first, and endorsing this belief was associated with increased willingness to be vaccinated. Vaccine ambivalence may in part have been shaped by the prevalence of misinformation and disinformation, as reflected by widespread agreement with negative statements about vaccine safety. Such findings highlight the public health importance of reducing and countering the dissemination of inaccurate information [33,47,48,75]. Recent research has identified associations between COVID-19-related misinformation and disinformation and ambivalence about COVID-19 vaccines [10,76,77,78]. Our findings suggest that those who were not currently willing to receive a COVID-19 vaccine may not have firmly fixed beliefs, indicating a potential to build vaccine confidence through continuing education from trusted sources.

Prior qualitative research among racially and ethnically diverse PWUD has found that the most common form of interpersonal discrimination experienced was as result of their drug-using status [79]. Stigma and discrimination based on identification as PWUD (either by self or by others) may reinforce medical mistrust. This mistrust may increase the susceptibility of PWUD to mis- and disinformation about COVID-19, resulting in missed opportunities to deliver prevention and education messages available in traditional healthcare settings [47,80]. Findings of high degrees of vaccine altruism and disproportionally low COVID-19 vaccine willingness suggest the potential for future research to better elucidate the relationship between the two, including the potential role of stigma and discrimination in willingness to receive COVID-19, potential concerns regarding interactions with the police when seeking vaccination, the influence of mis- or disinformation on the internet or social media, or health care system barriers such as requiring identification or co-pay for vaccine receipt.

Almost half of participants in our survey reported receiving most of their COVID-19 information from media sources, including social media and TV, both of which are sources that have been associated with mis- and disinformation about COVID-19 [75,76,81,82,83]. More than 70% of participants reported low or no confidence in the COVID-19 information they received from these sources. In contrast, while fewer than 20% of participants reported receiving COVID-19 information from healthcare providers, a majority reported trusting the COVID-19 information they received from healthcare providers, suggesting a need to improve the flow of COVID-19 vaccination information from healthcare providers to their patients. Further, 75% reported past 30-day use of harm reduction or substance use disorder (SUD) programs, and of these, 89% and 79% respectively considered them trusted sources of COVID-19 information. These findings indicate the potential role these harm programs can play in providing trusted COVID-19 information as well as the potential for these sites to serve as vaccination sites themselves or to offer effective linkages to vaccination sites.

In the US, inequities in geospatial COVID-19 vaccine access have been shown to vary by race/ethnicity as well as factors at the area level (e.g., neighborhood poverty) [84,85,86]. Factors plausibly related to vaccine access, such as distance from vaccine sites or consistency of internet access, often vary by geography, including within NYC, with these disparities overlapping with the areas where our survey participants were recruited for interviews [85,87]. The degree of COVID-19 vaccine willingness in our sample was similar to findings from other studies of contemporaneous general US adult populations, in which COVID-19 vaccine willingness ranged from 40–80% [88,89,90]. This suggests that PWUD may not be more difficult to engage with respect to COVID-19 vaccination than the general adult population. Further study is needed to examine whether living in areas with disparate access to vaccines may adversely impact the perceptions of COVID-19 risk, the benefit of vaccination, or the degree of medical mistrust. Ensuring geospatial equity in COVID-19 vaccine access in both traditional (e.g., primary care clinics) and non-traditional (e.g., harm reduction programs) settings is essential to achieving high vaccination rates and preventing morbidity and mortality among PWUD [91,92].

The ambivalence and medical mistrust toward COVID-19 vaccines suggests a potential role for motivational interviewing techniques in order to elicit and resolve identified ambivalence and mistrust [93,94]. Motivational interviewing has been identified as an effective individual-level intervention to address similar instances of ambivalence and mistrust concerning the safety, efficacy, and community benefit of health interventions regarding HIV treatment. Moreover, interventions that directly address medical mistrust more generally and mistrust rooted in historical racism specifically have shown promise in engaging diverse groups in HIV testing and treatment [95,96].

Our findings suggest that strategies to enhance COVID-19 vaccine uptake among PWUD need to recognize ambivalence and leverage altruism for this population. Further, there is a need for vaccination services to be delivered in a way that fosters trust, recognizes the intersectional identities of PWUD, and acknowledges and works to minimize stigma. As other studies have found, vaccine uptake and greater population-level immunity could potentially be achieved through a combination of individual- and social-network messaging and low-threshold vaccine access in non-stigmatizing settings, such as harm reduction programs and SSPs, delivered by trusted and respected people [9,33,44]. Additionally, in November 2021, NYC opened the first publicly recognized overdose prevention centers (OPCs) in the United States; OPCs are designed to reduce overdose mortality by providing a safe space for drug consumption. OPCs hold promise for the delivery of additional public health services such as COVID-19 vaccination [97].

### Limitations

Our study has several limitations. Both the epidemiology and scientific understanding of and the interventions available for COVID-19 changed rapidly during the initial months of the pandemic. Consequently, any assessment of perceptions reflects the specific time period in which the study was conducted. This was a cross-sectional study at one point in time, and PWUD perceptions may have changed. Small sample sizes precluded a number of subgroup analyses, and led to the reliance of bivariate analyses, which may be subject to type I error. Our study employed a convenience sampling frame, which was not representative of all PWUD, as eligibility criteria excluded PWUD who had already received any COVID-19 doses. In addition, the study was conducted in NYC and may not represent PWUD in other geographic locations, particularly non-urban areas.

## 5. Conclusions

This study, and the contributions of the participants, identified many important potential opportunities for public health to improve COVID-19 vaccine confidence and uptake among PWUD. The findings highlight that COVID-19 vaccine ambivalence expressed by PWUD may in part result from unaddressed questions and concerns which may be amendable to intervention through non-stigmatizing and respectful discussion. Altruistic beliefs and the importance of community were prevalent among participants; public health interventions and messaging should incorporate these values in order to increase vaccine uptake. Further, engaging PWUD in evidence-based, scientifically accurate, and non-stigmatizing discussions, interventions, and services at venues PWUD frequent and trust are needed to enhance COVID-19 vaccine acceptance and improve COVID-19 vaccination rates.

## Figures and Tables

**Table 1:** Characteristics and associations with willingness to receive COVID-19 vaccine among people who use drugs in NYC, 2021.

	Overall Sample			Willingness to Receive COVID-19 Vaccine						
*Characteristic*	n ^1,2^	Percent ^3^	95% CI	Yes, n = 38	Yes, %	95% CI	No, n = 62	No, %	95% CI	*p*-Value
**People who use illicit drugs (past 30 days)**	100	100%		38	38%	37–39%	62	62%	61–63%	
**Borough of interview**										
Bronx	46	46%	37–58%	20	53%	37–68%	26	42%	30–54%	0.635
Brooklyn	4	4%	12–10%	1	3%	0–13%	3	5%	2–13%	
Manhattan	22	22%	15–31%	7	18%	9–33%	15	24%	15–36%	
Queens	24	24%	17–33%	7	18%	9–33%	17	27%	18–40%	
**Borough of residence**										
Bronx	30	30%	22–40%	15	39%	26–55%	15	24%	15–36%	0.514
Brooklyn	16	16%	10–24%	6	16%	7–30%	10	16%	9–27%	
Manhattan	24	24%	17–33%	8	21%	11–36%	16	26%	17–38%	
Queens	19	19%	13–28%	6	16%	7–30%	13	21%	13–32%	
**Gender**										
Male	80	80%	71–87%	32	84%	70–93%	48	77%	66–86%	0.268
Female	19	19%	13–28%	5	13%	6–27%	14	23%	14–34%	
**Age, mean in years (SD)**	45 (10)	n/a	n/a	38	47 (9)	n/a	62	43 (10)	n/a	0.024 ^6^
**Age category, in years**										
18–29	6	6%	3–12%	1	3%	0–13%	5	8%	3–18%	0.096 ^7^
30–39	26	26%	18–35%	5	13%	6–27%	21	34%	23–46%	
40–49	24	24%	17–33%	10	26%	15–42%	14	23%	14–34%	
50–59	40	40%	31–50%	20	53%	37–68%	20	32%	22–45%	
60–69	4	4%	2–10%	2	5%	1–17%	2	3%	1–11%	
**Race/Ethnicity**										
non-Hispanic Black	22	22%	15–31%	11	29%	17–45%	11	18%	10–29%	0.384 ^8^
non-Hispanic White	30	30%	22–40%	9	24%	13–39%	21	34%	23–46%	
Other	4	4%	2–10%	2	5%	1–17%	2	3%	1–11%	
Hispanic	44	44%	35–54%	16	42%	28–58%	28	45%	33–57%	
*Puerto Rican*	30	68%	20–47%	12	75%	50–90%	18	64%	46–79%	
*Other Hispanic ancestries*	14	32%	53–80%	4	25%	10–49%	10	36%	21–54	
**Education**										
Up to 8th grade	11	11%	6–19%	2	5%	1–17%	9	15%	8–25%	0.204 ^8^
Some high school	27	27%	19–36%	11	29%	17–48%	16	26%	16–38%	
High school diploma or GED	39	39%	30–49%	14	37%	23–53%	25	3%	29–53%	
Associate’s degree or some college	17	17%	11–26%	8	21%	11–36%	9	15%	8–25%	
Bachelor’s degree	4	4%	2–10%	2	5%	1–17%	2	3%	0–11%	
Master’s, professional or doctoral degree	1	1%	0–5%	1	3%	0–13%	0	0%	n/a	
**Services accessed (past 30 days) ^4^**										
MOUD	41	41%	32–51%	24	63%	40–70%	17	27%	18–40%	n/a
Primary care	26	26%	18–35%	11	29%	17–48%	15	24%	15–36%	
HIV care	8	8%	4–15%	3	8%	3–21%	5	8%	3–18%	
SSP	33	33%	25–43%	13	34%	21–50%	20	32%	22–45%	
Other	12	12%	7–20%	7	18%	9–33%	5	8%	3–18%	
**Injection drug use (past 30 days)**										
Yes	47	47%	38–57%	21	55%	40–70%	26	42%	30–54%	0.221
No	52	52%	42–62%	17	45%	30–60%	35	56%	44–68%	
**Consistent internet access**										
Yes	65	65%	55–74%	23	61%	45–74%	42	68%	55–78%	0.374
No	34	34%	25–44%	14	37%	23–53%	20	32%	22–45%	
**Had a job that required in-person contact ^5^**										
Yes	31	31%	23–41%	9	24%	13–39%	22	35%	25–48%	0.268
No	66	66%	56–75%	26	68%	53–81%	13	21%	13–33%	

Abbreviations: medication for opioid use disorder (MOUD), syringe service program (SSP), general educational development (GED), standard deviation (SD). ^1^ n and % unless otherwise noted, missing data were <5%. ^2^ *p*-values were calculated from Chi-Square tests, for calculations where one or more cells were fewer than 10 a Fisher’s Exact test was calculated. ^3^ Column percents are calculated. All percentages are out of 100 reflecting the total number of participants interviewed. In the case that percentages do missing data.not add to 100 they reflect. ^4^ Not mutually exclusive. ^5^ Those who were employed in jobs that required in-person contact were compared to those who were unemployed or whose jobs did not require in-person contact. ^6^ An ANOVA test was conducted given the continuous variable of age. ^7^ This is the *p*-value for the association with age in 5 categories. For the association of age dichotomized at < or ≥40 years the *p*-value was <0.001. ^8^ This is the *p*-value for the binary comparison of those receiving education through high school compared to those with some college or more.

**Table 2:** Experiences and perceptions of vaccination, and associations with willingness to receive COVID-19 vaccine, among people who use drugs in NYC, 2021.

				Willingness to Receive COVID-19 Vaccine						
	n = 100 ^1^	% ^1^	95% CI	Yes n (n = 38)	Yes, % ^1^	95% CI	No n (n = 62)	No, % ^1^	95% CI	*p*-Value ^2^
**Friend or family diagnosed with COVID-19**										
Yes	41	41%	32–51%	22	58%	42–72%	37	60%	47–71%	0.860
No	59	59%	49–68%	16	42%	28–58%	25	40%	29–53%	
**Thought COVID-19 most likely originated in a lab**										
Yes	67	67%	57–75%	20	53%	37–68%	47	76%	64–85%	**0.017**
No	33	33%	25–43%	18	47%	32–63%	15	24%	15–36%	
**Attempted to make COVID-19 vaccine appointment**										
Yes	23	23%	16–32%	17	45%	30–60%	6	10%	5–20%	**<0.001**
No	77	77%	68–84%	21	55%	40–70%	56	90%	80–95%	
**Confidence level in decision about willingness to be COVID-19 vaccinated**										
Very confident ^3^	70	70%	60–78%	27	71%	55–83%	43	69%	57–36%	0.857
Not very confident	30	30%	22–40%	11	29%	17–45%	19	31%	21–43%	
**Having a COVID-19 vaccine paused (n = 79)**										
Would affect decision to be COVID-19 vaccinated	58	73%	48–67%	17	57%	30–60%	41	84%	71–91%	**<0.001**
Would not affect decision to be COVID-19 vaccinated	21	27%	14–30%	13	43%	21–50%	8	16%	9–29%	
**Having a COVID-19 vaccine reauthorized (n = 87)**										
Would affect decision to be COVID-19 vaccinated	64	74%	54–73%	21	62%	40–70%	43	81%	69–89%	**0.046**
Would not affect decision to be COVID-19 vaccinated	23	26%	16–32%	13	38%	21–50%	10	19%	11–31%	
**Received other adult vaccinations**										
Yes	76	76%	67–83%	30	79%	64–89%	46	74%	62–83%	0.59
No	24	24%	17–33%	8	21%	11–36%	16	26%	17–38%	
**Had concerns about how fast the COVID-19 vaccine was made and released**										
Agree ^4^	74	74%	65–82%	26	68%	53–81%	48	77%	66–86%	0.319
Disagree	26	26%	18–35%	12	32%	19–47%	14	23%	14–34%	
**Would feel more comfortable receiving the COVID-19 vaccine if other respected people received it first**										
Agree	65	65%	55–74%	29	78%	63–89%	36	58%	46–70%	**0.039**
Disagree	34	34%	28–41%	8	22%	11–37%	26	42%	30–54%	

**Abbreviations: people who use drugs (PWUD).** ^1^ Total n = 100. Column percentages are calculated. Percentages that do not add to 100 reflect missing data. Missing data were <5% for any given variable. ^2^ *p*-values were calculated from Chi-Square tests, for calculations where one or more cells were fewer than 10 a Fisher’s Exact test was calculated. ^3^ Category of very confidence reflects response of being very confident in decision to be COVID-19 vaccinated or not; not very confident includes all other responses: kind of confdient, not very confident, and neutral. ^4^ The response ‘agree’ includes ‘strongly agree’, ‘agree’ and ‘neither agree nor disagree’ and the response ‘disagree’ includes ‘disagree’ and ‘strongly disagree’. For each dichtomoized variable the statisical significance did not change if ‘neither agree nor disagree’ was added to the ‘disagree’ repsonse or if it was added to the ‘agree’ response.

**Table 3:** Vaccine-related trust statements and associations with willingness to receive COVID-19 vaccine, among people who use drugs in NYC, 2021.

				Willingness to Receive COVID-19 Vaccine						
	n = 100 ^1^	% ^1^	95% CI	Yes n (n = 38)	Yes, % ^1^	95% CI	No n (n = 62)	No, % ^1^	95% CI	*p*-Value ^2^
**Vaccines are important for the health of my community**										
Agree ^3^	83	83%	74–89%	36	95%	83–99%	47	76%	63–85%	**0.015**
Disagree	15	15%	9–23%	4	11%	4–24%	11	18%	10–29%	
**My getting vaccinated for COVID-19 is important for the health of others in my community**							.			
Agree	82	82%	73–88%	36	95%	83–99%	46	74%	62–83%	**0.014**
Disagree	18	18%	12–27%	2	5%	1–17%	16	26%	17–38%	
**Vaccine safety data is often fabricated**										
Agree	67	67%	57–75%	18	47%	32–63%	49	79%	67–87%	**0.001**
Disagree	33	33%	25–43%	20	53%	37–68%	13	21%	13–33%	
**Immunizing children is harmful, and this fact is covered up**										
Agree	64	64%	54–73%	17	45%	30–60%	47	76%	64–85%	**0.002**
Disagree	18	18%	12–27%	14	37%	23–53%	4	6%	3–15%	
**People are deceived about vaccine efficacy**										
Agree	76	76%	67–83%	22	58%	42–72%	54	87%	77–93%	**0.001**
Disagree	24	24%	17–33%	16	42%	28–58%	8	13%	7–23%	
**Vaccine efficacy data is often fabricated**										
Agree	70	70%	60–78%	20	53%	37–68%	50	82%	71–90%	**0.002**
Disagree	29	29%	21–39%	18	47%	32–63%	11	18%	10–29%	
**People are deceived about vaccine safety**										
Agree	77	77%	68–84%	22	58%	42–72%	55	89%	78–94%	**0.001**
Disagree	23	23%	16–32%	16	42%	28–58%	7	11%	6–22%	
**The government is trying to cover up the link between vaccines and autism**										
Agree	74	74%	64–82%	22	59%	42–72%	52	84%	73–91%	**0.007**
Disagree	25	25%	18–34%	15	41%	26–55%	10	16%	9–27%	
**New vaccines carry more risks than older vaccines**										
Agree	75	75%	66–82%	26	68%	53–81%	49	80%	67–87%	0.179
Disagree	24	24%	17–33%	12	32%	19–47%	12	20%	11–31%	
**I am concerned about serious adverse effects of COVID-19 vaccine**										
Agree	83	83%	74–89%	28	74%	58–85%	55	89%	78–94%	**0.052**
Disagree	17	17%	11–26%	10	26%	15–42%	7	11%	18–40%	
**I am confident that the COVID-19 vaccine is safe for people of my race/ethnicity**										
Agree	80	80%	71–87%	36	95%	83–99%	44	71%	59–81%	**0.004**
Disagree	20	20%	13–29%	2	5%	1–17%	18	29%	19–41%	
**I am confident that people of my race/ethnicity will have equal access to the COVID-19 vaccine compared to other race/ethnicities**										
Agree	73	73%	64–81%	31	84%	67–91%	42	68%	55–78%	0.079
Disagree	26	26%	18–35%	6	16%	7–30%	20	32%	22–44%	
**I am confident that the COVID-19 vaccine is safe for people who use drugs**										
Agree	78	78%	69–85%	36	95%	83–99%	42	68%	55–78%	**0.002**
Disagree	22	22%	15–31%	2	5%	1–17%	20	32%	22–44%	
**I am confident that PWUD will have equal access to the COVID-19 vaccine compared to people who do not use drugs**										
Agree	42	42%	33–52%	14	37%	23–53%	28	45%	33–57%	0.413
Disagree	58	58%	48–67%	24	63%	47–77%	34	55%	43–67%	

Abbreviations: people who use drugs (PWUD). ^1^ Total n = 100. Column percentages are calculated. Percentages that do not add to 100 reflect missing data. Missing data were <5% for any given variable. ^2^ *p*-values were calculated from Chi-Square tests, for calculations where one or more cells were fewer than 10 a Fisher’s Exact test was calculated. ^3^ The response ‘agree’ includes ‘strongly agree’, ‘agree’ and ‘neither agree nor disagree’ and the response ‘disagree’ includes ‘disagree’ and ‘strongly disagree’. For each dichtomoized variable the statisical significance did not change if ‘neither agree nor disagree’ was added to the ‘disagree’ repsonse or if it was added to the ‘agree’ response.

## Data Availability

Not applicable.
